# Remote ischemic conditioning plus stenting for symptomatic carotid artery stenosis (RICSICAS): rationale and design

**DOI:** 10.3389/fneur.2026.1915534

**Published:** 2026-09-30

**Authors:** Jing Qiu, Wei Li, Yong-Gang Zhao, Jia-Qi Wang, Lu Wang, Hui-Sheng Chen

**Affiliations:** Department of Neurology, General Hospital of Northern Theater Command, Shenyang, China

**Keywords:** carotic artery, ischaemic conditioning, outcome, protocol, stroke

## Abstract

**Background:**

Remote ischemic conditioning (RIC) has emerged as a promising non-invasive strategy to protect the brain, with evidence suggesting its benefit in patients with carotid artery stenting (CAS). However, the long-term benefit and safety of chronic RIC in this population remain unknown.

**Objective:**

To evaluate whether chronic RIC reduces the incidence of major vascular events in high-risk patients with carotid artery stenosis who received CAS.

**Methods:**

RICSICAS is a prospective, randomized, open-label, blinded endpoint evaluation (PROBE), single-center trial. We plan to enroll 450 high-risk patients with symptomatic carotid artery stenosis (Essen score ≥ 3) scheduled for CAS. Participants will be randomly assigned (1:1) to receive either standard medical therapy plus RIC or standard medical therapy alone. The RIC protocol involves bilateral upper-arm cuff inflation to 200 mmHg (5-min inflation/5-min deflation, 5 cycles) performed 1–2 times daily, starting 3 days before CAS and continuing for 12 months post-procedure, thus constituting a combined peri-procedural and chronic RIC strategy. The primary efficacy endpoint is the composite of stroke, myocardial infarction, TIA, and vascular death within 12 months. Safety endpoints include any RIC-related adverse event (e.g., limb pain, petechiae, peripheral nerve injury), and any serious adverse event.

**Sample Size:**

Assuming a 12-month event rate of 20% in the control group and a 40% relative risk reduction with RIC, with 80% power and a two-sided α of 0.05, 355 patients are required. Accounting for a 20% dropout rate/loss-to-follow-up, as well as the consumption of the interim analysis, the total sample size is set as 450.

**Discussion:**

This trial will provide high-level evidence on the role of long-term RIC as an adjunctive therapy for high-risk patients with symptomatic carotid artery stenosis undergoing CAS.

**Clinical Trial Registration:**

Clinicaltrials.gov, identifier: NCT07412249.

## Introduction and rationale

Acute ischemic stroke remains a leading cause of death and disability worldwide and in China (1). Carotid artery stenosis is a pivotal etiology, responsible for a significant proportion of ischemic events (2). For patients with symptomatic stenosis, carotid revascularization via endarterectomy or stenting is a cornerstone of secondary prevention. However, carotid artery stenting (CAS), while minimally invasive, carries inherent risks. Several landmark randomized controlled trials have established that the periprocedural risk of stroke or death following CAS in symptomatic patients ranges from 6.8% to 9.6% at 30 days (3–5). Specifically, the Stent-Protected Angioplasty vs. Carotid Endarterectomy (SPACE) trial reported a 30-day rate of ipsilateral ischemic stroke or death of 6.8% (3); the Endarterectomy vs. Stenting in Patients with Symptomatic Severe Carotid Stenosis (EVA-3S) trial reported a rate of any stroke or death of 9.6% (4); and the International Carotid Stenting Study (ICSS) reported a rate of stroke, death, or procedural myocardial infarction of 8.5% (5). Beyond clinical events, the ICSS imaging substudy further revealed that periprocedural diffusion-weighted imaging (DWI) detects new ischemic brain lesions in a substantial number of patients, underscoring the embolic potential of the procedure (6). Furthermore, even with successful revascularization and optimal medical management, the long-term risk of stroke recurrence and other vascular events in high-risk patients remains clinically significant (7–9).

Remote ischemic conditioning (RIC) is an adaptive response whereby brief, repetitive cycles of ischemia and reperfusion in one vascular territory (e.g., a limb) confer protection against subsequent, more prolonged ischemia in a distant organ (e.g., the brain). The protective mechanisms are multifaceted, believed to involve humoral, neuronal, and systemic pathways leading to enhanced collateral circulation, anti-inflammatory effects, and improved endothelial function (10, 11). Its clinical translation has gained momentum. The RICAMIS trial demonstrated that RIC improved functional outcomes in patients with acute moderate ischemic stroke (12). Similarly, the RICA trial showed that chronic RIC reduced vascular events in patients with symptomatic intracranial atherosclerotic stenosis (13). Our prior mechanistic study (RIC-CCT) provided evidence that RIC can improve cerebral hemodynamics in severe carotid stenosis (14). Most pertinently, a proof-of-concept randomized trial by Zhao et al. provided initial evidence that RIC administered before CAS could reduce the volume and incidence of new DWI lesions (15). This study, however, was limited by short-term imaging endpoint.

Despite these promising signals, whether sustained RIC treatment translates into a reduction in hard clinical endpoints (such as stroke, myocardial infarction, and death) in this high-risk population remains unknown. We hypothesize that in patients with high-risk symptomatic carotid stenosis (Essen score ≥ 3) (8, 16), a regimen combining short-term pre-procedural RIC (to mitigate periprocedural injury) with prolonged post-procedural RIC (to promote vascular stability and long-term protection) will safely reduce the composite risk of major vascular events within one year following CAS.

In this context, the RICSICAS trial was designed to evaluate the effect of sustained RIC in high-risk symptomatic carotid artery stenosis patients scheduled to undergo CAS.

## Methods

### Study design

RICSICAS is a prospective, randomized, open-label, blinded endpoint evaluation (PROBE), single-center superiority trial. This protocol is reported in accordance with the SPIRIT 2013 Statement (Standard Protocol Items: Recommendations for Interventional Trials). The study flow is summarized in Figure 1.

**Figure 1:**
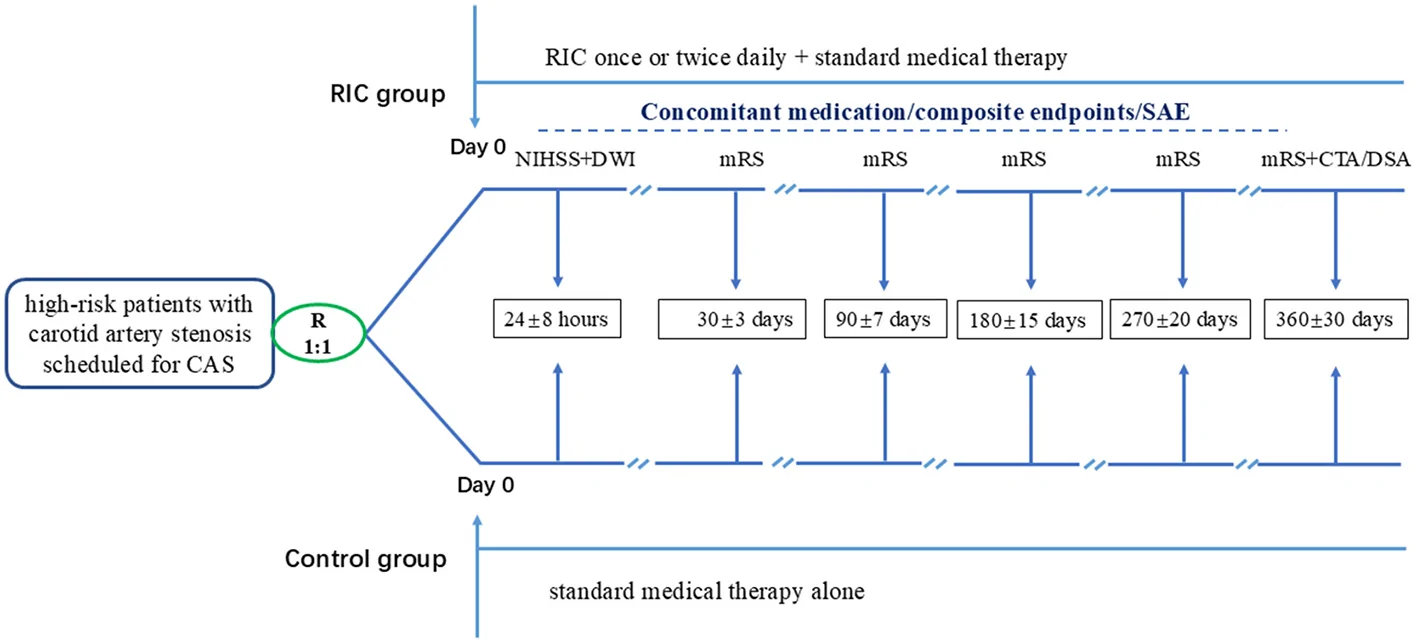
Study flowchart. CAS, carotid artery stenting; RIC, remote ischemic conditioning; NIHSS, National Institutes of Health Stroke Scale; MRI, magnetic resonance imaging; SAE, severe adverse event(s).

### Study participants

In this trial, eligible patients are adults with symptomatic, moderate-to-severe atherosclerotic stenosis (≥50% per NASCET criteria) of the internal carotid artery scheduled to undergo CAS. Key exclusion criteria include non-atherosclerotic stenosis, contraindications to RIC, history of intracranial hemorrhage (within 90 days), intracranial tumor, aneurysm, or arteriovenous malformation. Patients will be enrolled at General Hospital of Northern Theater Command between March 2026 to September 2027. The detailed inclusion and exclusion criteria are shown in Table 1.

**Table 1:** The inclusion/exclusion criteria.

Inclusion criteria
• Age ≥ 40 years old;
• Patients with symptomatic moderate to severe stenosis of the internal carotid artery;
• History of ipsilateral cerebral ischemic symptoms within the past 180 days;
• Planned for carotid artery stent;
• Essen Score ≥ 3;
• Modified Rankin Scale score ≤ 1;
• Signed informed consent by patient or their legally authorized representative.
Exclusion criteria
• Uncontrolled severe hypertension (systolic blood pressure > 180 mmHg or diastolic blood pressure > 110 mmHg despite medication);
• Subclavian artery stenosis ≥ 50% or presence of subclavian steal syndrome;
• Severe hematological disorders or significant coagulation abnormalities;
• Contraindications to remote ischemic conditioning, such as severe soft tissue injury, fracture, or vascular injury in the upper limbs, or peripheral vascular disease in the distal upper limbs;
• Severe comorbid conditions with a life expectancy of less than 1 year;
• Participation in another clinical trial within the past 3 months or ongoing participation;
• Any other circumstances deemed by the investigator as unsuitable for participation in this clinical study.

### Standard protocol approvals, registrations, and patient consents

The RICSICAS trial was prospectively registered on ClinicalTrials.gov (NCT07412249). Ethical approval for the study protocol and procedures was granted by the Ethics Committee of the General Hospital of Northern Theater Command (IRB: Y (2026) 14). Prior to any study procedures, written informed consent will be secured from every participant or their legally authorized representatives.

### Randomization and blinding

Eligible participants will be randomized in a 1:1 ratio to the RIC group or the Control group. Randomization will be performed using a centralized, computer-generated block randomization sequence, stratified by Essen score (3–5 vs. 6–9) and age (< 65 vs. ≥65). The nature of intervention precludes blinding of patients and treating physicians. However, critical endpoint assessments will be conducted by assessors blinded to treatment allocation (blinded endpoint evaluation). All imaging analyses (MRI, CTA) will be performed by a core imaging laboratory whose staff are blinded to clinical data and group assignment.

### Interventions

In RIC Group, patients will undergo RIC using an automated device, in addition to standard therapy. The protocol consists of five cycles of bilateral upper-arm cuff inflation to 200 mmHg for 5 min, each followed by 5 min of deflation. This session will be performed once or twice daily, at the patient's discretion, to optimize long-term adherence over the 12-month period. The electronic device will automatically log the date, time, and number of completed cycles, enabling precise quantification of the cumulative RIC dose. In Control Group, patients will receive standard medical therapy alone, without any sham RIC procedure.

All patients will receive guideline-directed standard medical therapy, including dual antiplatelet therapy (aspirin 100 mg + clopidogrel 75 mg daily) peri-procedurally, transitioning to long-term single antiplatelet therapy, statin, and aggressive management of vascular risk factors.

### Study procedures and follow-up

Assessments will occur at the following time points: Screening/Baseline, 24 h post-stenting, and at 1, 3, 6, 9 and 12 months post-randomization (Table 2). Key assessments include:

- **Clinical:** NIHSS, mRS, collection of adverse events and concomitant medications.
- **Imaging:** Brain MRI (including DWI and FLAIR sequences) at baseline and 12 months; CT/CTA of the cervical and cerebral arteries at baseline and 12 months; optional optical coherence tomography (OCT) during the stenting procedure and at 12-month follow-up for a subgroup.
- **Laboratory:** Routine blood tests, biomarkers (e.g., hs-CRP, VEGF), and visceral organ function tests (renal, pancreatic, cardiac).
- **Physiological:** Assessment of cerebral autoregulation via transcranial Doppler at baseline and 12 months.

**Table 2:** Timing of all procedures.

Timepoint	Screening	0	24 ±8 hours	30 ±3 days	90 ±7 days	180 ±15 days	270 ±20 days	360 ±30 days
Eligibility screen	x							
Informed consent	x							
Randomization	x							
Head CT/MRI/DSA	x		x^1^					x^2^
Essen assessment	x							
NIHSS assessment	x	x	x					
mRS assessment	x			x	x	x	x	x
Medical history	x							
Population characteristics	x							
Vital signs	x	x	x	x	x	x	x	x
Blood routine	x							
Urinalysis	x							
Coagulation test	x							
Serum biochemistry	x							
ECG	x							
Concomitant medication		x	x	x	x	x	x	x
Adverse events		x	x	x	x	x	x	x
Stroke and other vascular events		x	x	x	x	x	x	x

CT indicates computed tomography; MRI, magnetic resonance imaging; NIHSS, National Institutes of Health Stroke Scale; mRS, modified Rankin Scale; ECG, electrocardiogram.

^1^ Diffused weighted imaging is mandatory.

^2^ CTA or DSA is mandatory.

### Outcomes

Primary efficacy endpoint is time to the first occurrence of any component of the composite endpoints (ischemic stroke, hemorrhagic stroke, myocardial infarction [excluding silent MI], TIA, or vascular death) within 12 months after randomization. Ischemic stroke is defined as acute focal neurological deficit lasting >24 h with imaging evidence of infarction, or lasting < 24 h with imaging confirmation; Hemorrhagic stroke is defined as acute focal neurological deficit with imaging evidence of intracranial hemorrhage; Myocardial infarction is defined according to the Third Universal Definition of Myocardial Infarction (elevated cardiac biomarkers + ischemic symptoms or ECG changes); TIA is defined as transient (< 24 h) focal neurological deficit without acute infarction on neuroimaging, confirmed by a neurologist; Vascular death is defined as death due to stroke, MI, or other vascular causes (including sudden death).

Secondary efficacy endpoints include: occurrence of contrast staining on brain computerized tomography at 1 h after CAS; occurrence of contrast staining on brain CT at 24 h after CAS; occurrence of new infarct on brain DWI at 24 h after CAS; occurrence of cerebral hyperperfusion syndrome (CHS) within 24 h after CAS; composite of death, stroke, or myocardial infarction; non-fatal stroke and TIA within 30 days; nonfatal myocardial infarction within 12 months; new silent cerebral infarcts on MRI at 12 months; ipsilateral (to the stented artery) nonfatal stroke and TIA within 12 months; degree of in-stent restenosis (≥50%) on CTA/DSA at 12 months; change in collateral circulation status (assessed by CTA or DSA) from baseline to 12 months; all-cause mortality within 12 months, and changes in mRS scores over time.

Safety endpoints include any RIC-related adverse event (e.g., limb pain, petechiae, peripheral nerve injury); any Serious Adverse Event (SAE).

Exploratory endpoints include changes in serum biomarkers, plaque characteristics on OCT, cerebral small vessel disease burden on MRI, and cerebral autoregulation parameters.

All primary and secondary endpoint events will be adjudicated by an independent Clinical Events Committee (CEC) whose members are blinded to treatment allocation. The CEC will review all source documentation (medical records, imaging, laboratory results) using predefined standardized criteria.

### Quality control, data management and monitoring

In this clinical trial, an independent Data and Safety Monitoring Committee (DSMC) will conduct safety data verification at six-month intervals. Once follow-up has been completed for 60% of participants, an interim analysis will be performed. Additionally, neuroimaging data will be collected centrally and independently assessed by two neuroradiologists. All study data will be recorded on paper Case Report Forms (CRFs). Subsequently, data will be transcribed into a secure electronic database using a double-entry verification process to ensure accuracy. An independent DSMB will be convened to review unblinded safety and efficacy data at regular intervals and for interim analysis. All SAEs must be reported to the DSMB and the relevant Ethics Committee within 24 h. An independent Data and Safety Monitoring Board (DSMB) will conduct safety data verification at six-month intervals. A prespecified interim analysis for efficacy will be performed when approximately 50% of the required primary events have been observed. The O'Brien-Fleming alpha-spending function will be used to preserve the overall two-sided Type I error at 0.05. The nominal significance thresholds are: Interim analysis: *p* < 0.003; Final analysis: *p* < 0.049. At the interim analysis, conditional power will be calculated. If conditional power is < 50%, the DSMB may recommend increasing the sample size (maximum 600 patients) based on the observed event rate and treatment effect, using the O‘Brien–Fleming boundary without further alpha adjustment. Enrollment will be stopped for efficacy if the *p*-value at the interim analysis crosses the specified threshold (*p* < 0.003).

### Sample size calculation

The calculation is based on the primary composite endpoint. In high-risk patients (Essen score ≥ 3) with symptomatic carotid artery stenosis undergoing CAS, the 1-year risk of major vascular events (stroke, myocardial infarction, TIA, or vascular death) is estimated at approximately 20%. This estimate is further supported by the REACH registry demonstrated a 1-year combined vascular event rate of 12.2% in Essen score ≥3 patients treated with standard medical therapy alone (8); among high risk patients undergoing CAS, the 1-year major adverse event rate ranges from 12% to 16% (9); and when periprocedural risks (6–12% at 30 days from major CAS trials (3–5)) are combined with the inherent risk of this high-risk population, the cumulative 1-year event rate approaches 25–30%. We hypothesize that RIC will reduce this rate by 40% (to 12%). Using a two-sided log-rank test with α = 0.05 and power = 80%, and the calculated sample size to test the superiority hypothesis is 355 patients. Anticipating a 20% dropout/loss-to-follow-up rate over 12 months, as well as the consumption of the interim analysis, we plan to enroll a total of 445 patients (225 per group).

### Statistical analysis plan

The primary analysis will follow the Intention-to-Treat (ITT) principle, including all randomized patients. A secondary per-protocol (PP) analysis will also be conducted, which will include only patients who achieved good adherence to the RIC protocol, predefined as completion of ≥70% of prescribed RIC sessions over the 12-month period. Time-to-event data for the primary endpoint will be analyzed using the Kaplan-Meier method, with between-group comparisons made using the log-rank test. The treatment effect will be quantified as a Hazard Ratio (HR) with a 95% confidence interval (CI) using a Cox proportional hazards model, adjusted for stratification factors. Secondary endpoints will be compared using binary logistic regression analysis (binary), general linear model (continuous), or the Kaplan-Meier survival curve (time to event). Ordinal analysis of the mRS will be performed using ordinal logistic regression. Further, the log-rank test will be used for comparison between groups. Hazard ratios and 95% confidence intervals will be estimated using the Cox proportional hazards model. A two-sided *P*-value < 0.05 will be considered statistically significant.

Multiplicity strategy: For the confirmatory primary endpoint, Type I error is controlled through the O'Brien-Fleming alpha-spending function as described above. For secondary and exploratory analyses, no formal multiplicity adjustment (e.g., Bonferroni) will be applied, to avoid overly conservative corrections that might obscure potentially meaningful signals. All secondary, subgroup, and exploratory analyses will be clearly flagged as unadjusted for multiplicity, and results will be reported with effect sizes, 95% confidence intervals, and nominal *p*-values, accompanied by explicit interpretative caveats. These analyses should be considered hypothesis-generating rather than confirmatory.

Missing data handling: Endpoints will be not required to impute. Variables included in the adjusted analysis will be imputed with simple method according to its characteristics. Continuous variables will be imputed with median values and Categorized variables will be imputed with mode values.

Censoring rules: Patients completing 12-month follow-up without an event will be administratively censored at the last contact date. Patients lost to follow-up will be censored at the date of last known contact. Patients withdrawing consent will be censored at the withdrawal date.

Assessment of proportional-hazards assumption: Formal testing: Grambsch-Therneau global test; Contingency plans: If the assumption is violated, we will report Restricted Mean Survival Time (RMST) as a complementary analysis (which does not require proportional hazards), and consider stratified Cox models or time-varying coefficient models if severe violation is detected.

Analysis of repeated mRS measurements: Generalized Estimating Equations (GEE) with cumulative logit link for ordinal mRS (0–6), including treatment, time, and treatment × time interaction as fixed effects, with an unstructured or exchangeable working correlation matrix selected by Quasi-Information Criterion.

All subgroup analyses are explicitly exploratory. Interaction will be tested by including treatment × subgroup product terms in the Cox model, with Wald test *p*-values reported. Prespecified subgroups (*n* = 4): age (< 65 vs. ≥65), stenosis severity (50–69% vs. 70–99%), Essen score (3–5 vs. 6–9), and diabetes (yes vs. no). To address the resulting heterogeneity in treatment exposure, we will perform prespecified secondary analyses stratifying patients by: (1) average daily session frequency (once vs. twice daily, defined as ≥70% of days at the respective frequency); and (2) cumulative total sessions completed over 12 months, analyzed as a continuous variable and by quartiles, or prespecified landmark analyses for early (0–30 days) and late (31–365 days) periods. These exploratory analyses will help clarify whether a dose-response relationship exists. We explicitly acknowledge the limited power for interaction tests given the sample size and will interpret even statistically significant interactions as hypothesis-generating only. Results will be presented as forest plots with treatment-effect estimates and 95% CIs within each subgroup, with interaction *p*-values provided as descriptive guidance rather than definitive evidence.

### Study organization and funding

The Trial Steering Committee (TSC), comprising external scientific advisors, will conduct regular oversight of the study conduct and data management. The TSC will hold meetings every six months, either via teleconference or in person, to provide recommendations pertaining to patient enrollment and trial implementation. This trial is sponsored and supported by the Cerebrovascular Disease Collaboration & Innovation Alliance (CDCIA) of Liaoning Province.

### Current status

At the time of this manuscript's initial submission, patient recruitment had commenced, with 55 patients enrolled. Recruitment will remain open until the target sample size is achieved, with completion anticipated no later than September 2027.

## Discussion

The RICSICAS trial addresses a significant unmet need in the management of high-risk symptomatic carotid stenosis. While CAS is effective, its therapeutic margin can be potentially widened by adjunctive neuroprotective strategies. RIC is an ideal candidate due to its non-invasive nature, excellent safety profile established in other settings, and pleiotropic mechanisms of action (10, 11) that align with the pathophysiology of both procedural embolization (3–6) and long-term atherosclerotic disease progression (7–9).

This trial builds upon the foundational work of Zhao et al. (15), but extends the investigation in crucial ways: by focusing on long-term clinical endpoints rather than short-term imaging surrogates, by employing a sustained RIC regimen spanning both the periprocedural and chronic phases, and by targeting a specifically defined high-risk population where the absolute benefit is likely to be greatest. The comprehensive assessment protocol, including advanced imaging and biomarkers, is designed not only to test the primary clinical hypothesis but also to elucidate the mechanistic pathways through which RIC may exert its effects in this population.

We acknowledge several limitations. First, our sample size calculation assumes a 40% relative risk reduction based on prior exploratory studies, which may be optimistic; an interim analysis will provide information to adjust the sample size if needed. Second, the open-label design without a sham procedure may introduce performance bias and affect subjective secondary outcomes (e.g., mRS), although the primary endpoints are objective vascular events adjudicated by blinded assessors. Third, long-term home-based RIC over 12 months poses adherence challenges. These will be mitigated by using a device with electronic adherence logging and by maintaining regular patient engagement during follow-up. We have predefined good adherence as completing at least 70% of prescribed sessions. In addition to the primary intention-to-treat analysis, we will perform per-protocol and sensitivity analyses to assess the impact of adherence on treatment effects.

In conclusion, RICSICAS is a pragmatic, yet mechanistic trial poised to determine whether long-term remote ischemic conditioning can improve outcomes after carotid stenting. A positive result would introduce a simple, safe, and cost-effective adjunctive therapy into clinical practice for a condition with substantial residual risk.
